# Microstructured Polymer Blend Surfaces Produced by Spraying Functional Copolymers and Their Blends

**DOI:** 10.3390/ma9060431

**Published:** 2016-05-31

**Authors:** Nelson Vargas-Alfredo, Juan Rodríguez Hernández

**Affiliations:** Instituto de Ciencia y Tecnología de Polímeros (ICTP-CSIC), C/Juan de la Cierva 3, Madrid 28006, Spain; nvaralf@hotmail.com

**Keywords:** spray deposition, polymer blends, amphiphilic copolymers, thermoresponsive surfaces, superhydrophobicity

## Abstract

We described the fabrication of functional and microstructured surfaces from polymer blends by spray deposition. This simple technique offers the possibility to simultaneously finely tune the microstructure as well as the surface chemical composition. Whereas at lower polymer concentration, randomly distributed surface micropatterns were observed, an increase of the concentration leads to significant changes on these structures. On the one hand, using pure homopolystyrene fiber-like structures were observed when the polymer concentration exceeded 30 mg/mL. Interestingly, the incorporation of 2,3,4,5,6-pentafluorostyrene changed the morphology, and, instead of fibers, micrometer size particles were identified at the surface. These fluorinated microparticles provide superhydrophobic properties leading to surfaces with contact angles above 165°. Equally, in addition to the microstructures provided by the spray deposition, the use of thermoresponsive polymers to fabricate interfaces with responsive properties is also described. Contact angle measurements revealed variations on the surface wettability upon heating when blends of polystyrene and polystyrene-*b*-poly(dimethylaminoethyl methacrylate) are employed. Finally, the use of spraying techniques to fabricate gradient surfaces is proposed. Maintaining a constant orientation, the surface topography and thus the contact angle varies gradually from the center to the edge of the film depending on the spray angle.

## 1. Introduction

The elaboration of functional microstructured interfaces is currently a center of intensive research. The large variety of applications derived from these materials include the design of superhydrophobic [1] surfaces, the design of surfaces for biomedical purposes [2], including tissue engineering [3], cell adhesion [4], or the elaboration of diffractive optical elements [5], just to mention few of them.

In order to fabricate functional microstructured surfaces, different approaches have been proposed including lithographic techniques or instability based approaches. For instance, the techniques grouped under the name of “soft lithography” have been proposed for the development of nano- and micro-structured organic and inorganic matter [6,7,8,9]. In recent years, many polymer-based microfabrication techniques [10] via microinjection molding [11,12], casting [5,13], and micro-hot embossing [14,15] have been developed. In contrast to the above mentioned methodologies, spray deposition, a commercial production for painting at low cost, has been successfully applied to prepare large area surface coatings on many different substrates. Sprayed coatings have found applications for instance in the fabrication of organic-based devices, such as transistors [16], solar cells [17] or sensors [18]. In these studies, spraying has been chosen due to its ability to prepare the large-area devices with low surface roughness.

In effect, most of the previous reports have been published to understand the fast kinetic process of spray deposition and evaluate the spraying conditions to produce rather homogeneous films [19,20]. However, more recently, studies carried out by Medeiros *et al.* [21] or Srinivasan and coworkers [22] showed that the use of this straightforward methodology permits the fabrication of microstructured interfaces [22] by formation of micrometer size fibers [21] or even to form highly hydrophobic surfaces by incorporation of fluorinated Polyhedral Oligomeric Silsesquioxane (POSS) nanoparticles on a polymer matrix [22].

Both studies provided evidence of the possibility of using this approach to obtain non-homogeneous films with a rather high surface roughness. In this manuscript, we aim to extend this concept and explore the fabrication of textured surfaces by spraying polymer blends with different functional copolymers.

In particular, we will first explore the incorporation of random copolymers either hydrophilic or hydrophobic functional groups in a polymer matrix. The effect of the concentration as well as the possibility to fabricate gradient surfaces is described as well. In this context, it is worth mentioning that a straightforward fabrication of gradient surfaces changing a particular property along a particular direction may find practical applications in multiple areas including cell-motility, diagnostics or nanotribology. Most of the strategies reported up to now that fabricate gradient surfaces resort to sophisticated systems including corona and plasma discharge, palladium deposition or gas diffusion technique, among others [23,24,25]. Herein, we aim to illustrate the possibility to produce, in one single step, surfaces with gradual topography by using spray deposition. In the last part of this manuscript, we studied the use of spray to fabricate more sophisticated surfaces, also known as “smart surfaces”, that respond to variation on the environmental temperature by using blends of a homopolymer and a thermoresponsive block copolymer.

## 2. Discussion

**Figure 1:**
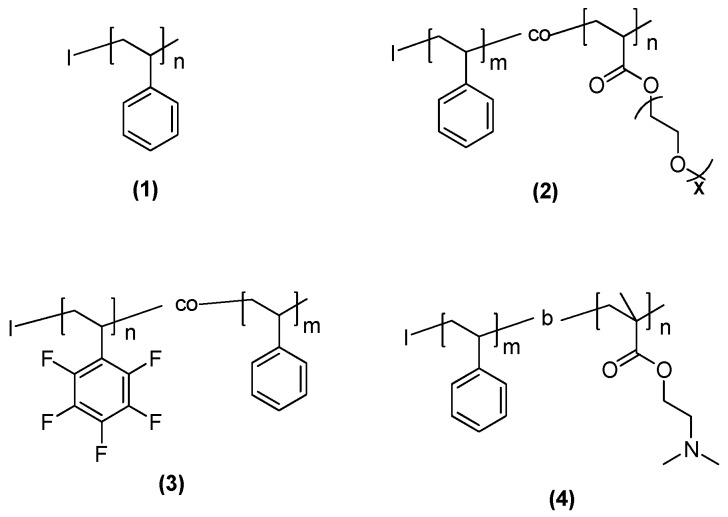
Chemical structures of the polymers employed in this study: (**1**) homopolystyrene; (**2**) polystyrene-*co*-poly(polyethylenglycol methacrylate) (PS-*co*-PPEGMA) (**3**) polystyrene-*co*-poly(2,3,4,5,6-pentafluorostyrene) (PS-*co*-P5FS) and (**4**) polystyrene-*b*-poly(dimethylaminoethyl methacrylate).

The fabrication of the functional surfaces was carried out using a polymer matrix, in this case a high molecular weight homopolystyrene (PS) blended with a variable amount of the different copolymers. For this study, we fabricated three different copolymers bearing hydrophilic or hydrophobic functional groups and also stimuli responsive properties as depicted in Figure 1. Amphiphilic copolymers were obtained either using poly(ethylenglycol methacrylate) (PEGMA) or dimethylamino ethyl methacrylate (DMAEMA) as comonomers. Moreover, double hydrophobic copolymers were obtained copolymerizing styrene (S) and 2,3,4,5,6-pentafluorostyrene (5FS). Moreover, the block copolymer prepared using DMAEMA, *i.e.*, polystyrene-*b*-poly(dimethylaminoethyl methacrylate) (PS-*b*-PDMAEMA) exhibits additionally pH and thermal response.

### 2.1. Surface Microstructures with Variable Wettability at Low Polymer Concentrations

**Figure 2:**
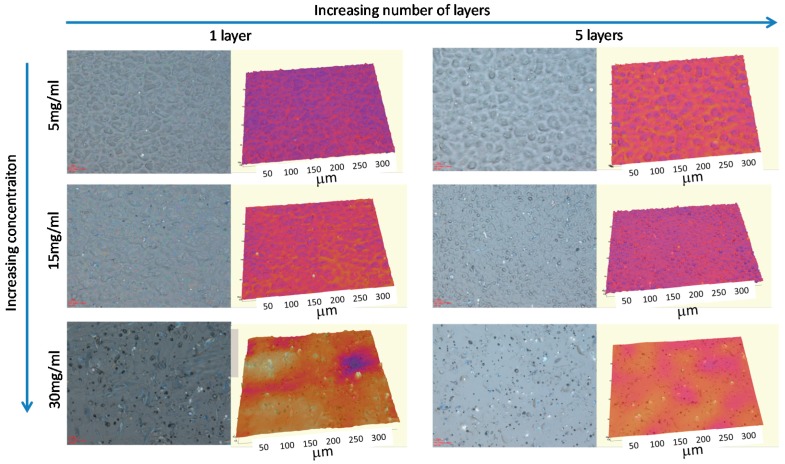
Optical microscope images and 3D optical profile images of the glass surfaces, sprayed using PS/CDCl_3_ solutions at different concentrations: 5, 15 and 30 mg/mL. In addition, either one or five layers were sprayed onto the surface.

As has been already evidenced by other authors, the solution concentration plays a major role in the formation of surface micro-textures during the spraying process [17,22]. Thus, the first series of experiments were conducted in a diluted concentration regime between 5 and 30 mg/mL. In addition, the number of layers was equally varied between one and ten. As made evident in Figure 2, using only homopolystyrene (PS), both concentration and the number of sprayed layers clearly induced the formation of different surface patterns. Lower concentrations produced microstructured patterns and average roughness R_a_ ~ 0.36 μm. An increase of the concentration of the solution employed up to 15 mg/mL produced a significant decrease of the surface roughness (R_a_ ~ 0.22 μm). Finally, spray carried out from 30 mg/mL solutions led to films with homogeneous smooth textures with low surface roughness R_a_ ~ 0.15 μm.

**Figure 3:**
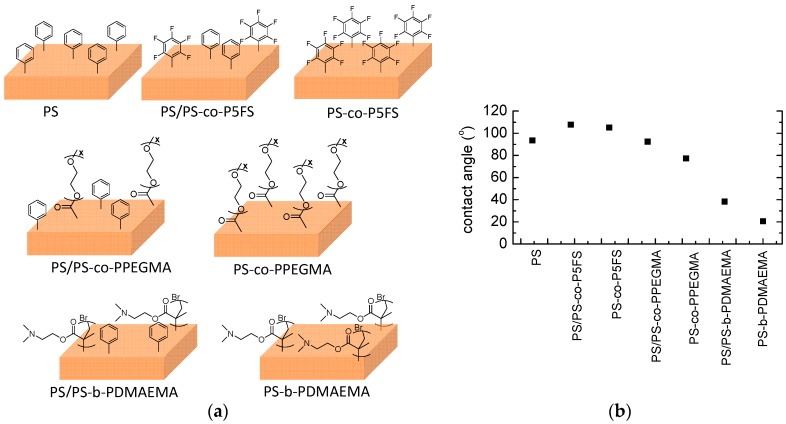
(**a**) Illustrative cartoon of the different surfaces explored prepared from either the pure polymers or blends; (**b**) Contact angle values of the surface prepared by spraying using polymer concentrations of 5 mg/mL and coating 10 layers.

In order to vary the functionality of the microstructures produced at low concentrations, blends with different copolymers having either hydrophilic or hydrophobic functional groups were fabricated. The surface structure obtained upon 10 layers using polymer solutions with a concentration of 5 mg/mL is depicted in Figure 3. Figure 3 also depicts the contact angle measured for all the blends as well as for the pure copolymers included for comparative purposes.

As expected, the incorporation of highly hydrophobic functional groups clearly increases the contact angle observed. Thus, an increase of around 20° was observed in blends of PS with PS-*co*-P5FS and when using exclusively PS-*co*-P5FS in comparison to pure PS. On the contrary, the incorporation of hydrophilic PEGMA or DMAEMA monomers significantly decreased the contact angle. In the case of PS-*co*-PPEGMA blended with PS, the contact angle remains similar to the values encountered for PS. Most probably, the low amount of PPEGMA within the total blend and the statistical structure of the amphiphilic copolymer did not permit considerable changes in the wettability. However, a larger increase of the surface wettability was observed in the case of the microstructured films produced from the pure PS-*co*-PPEGMA copolymers with contact angle values below 80°.

Interestingly, the block copolymer structure in the PS-*b*-PDMAEMA appeared to play a role in the surface wettability. In comparison with the PS-*co*-PPEGMA, the amount of the hydrophilic part is significantly lower, *i.e.*, 35 mol % in the statistical copolymer *versus* a 27 mol % in the block copolymer. Nevertheless, for similar blends, the incorporation of the block copolymer reduces the contact angle to a larger extent. More precisely, blends of PS/PS-*b*-PDMAEMA exhibit contact angles of around 35°, whereas blends of PS/PS-*co*-PPEGMA produced surfaces with contact angles of around 85°. Finally, the pure copolymer produces more hydrophilic surfaces (contact angles below 25°).

### 2.2. Functional Surface Microstructures Polymer Concentrations above 30 mg/mL

**Figure 4:**
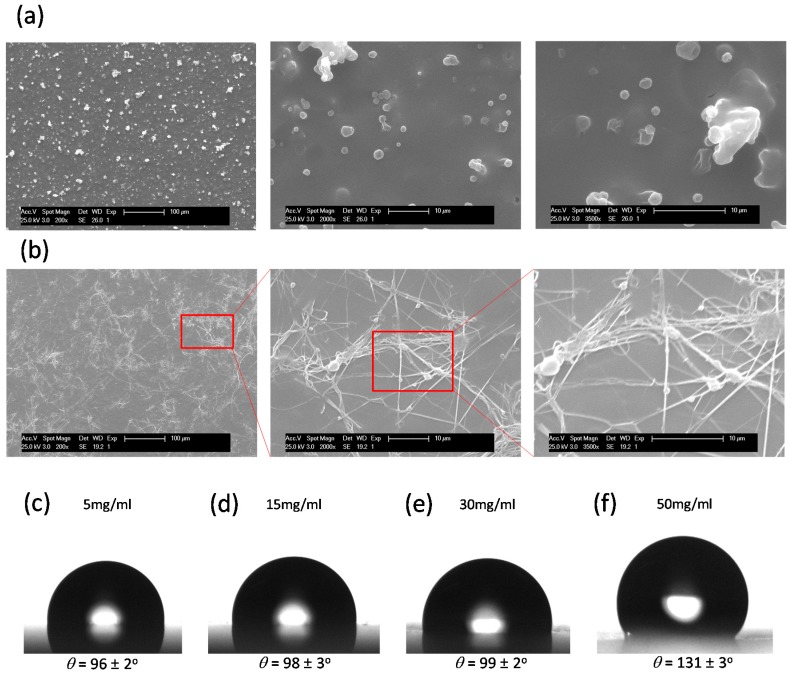
SEM images of the PS_40_ (**a**) and PS_250_ (**b**) surfaces prepared at a concentration of 50 mg/mL. Contact angle values measured for PS_250_ sprayed surfaces using polymer solutions at different concentrations: (**c**) 5 mg/mL; (**d**) 15 mg/mL; (**e**) 30 mg/mL and (**f**) 50 mg/mL. The films were fabricated applying 10 layers.

In the concentration regime depicted above, the surfaces exhibited randomly oriented micropatterns. However, an increase of the polymer concentration above 30 mg/mL induced interesting changes on the surface topography that largely depend on the molecular weight of the polymer employed. In the case of PS_40_ spraying, using high concentrations produce surfaces with micrometer size features (Figure 4a). On the other hand, when using PS_250_, an increase of the surface concentration leads to submicrometer (200–400 nm) fiber like structures (Figure 4b). These variations require further investigation but could be, in principle, associated with differences in the solution viscosity. Interestingly, the formation of fibers has been associated with an increase of the contact angle in comparison to previous textured surfaces prepared at lower concentrations. As a result, as depicted in Figure 4c–e, whereas the contact angles remain constant with values of around 96°–99° for the range of concentrations between 5 and 30 mg/mL, the surfaces prepared from 50 mg/mL solutions have contact angles above 130°. Similar observations were reported by Srinivasan *et al.* using POSS/Polymethyl methacrylate (PMMA blends [22]). However, in their study, the highly hydrophobic structure of the POSS employed together with the fiber-like structure was responsible for the larger increase of the contact angle.

**Figure 5:**
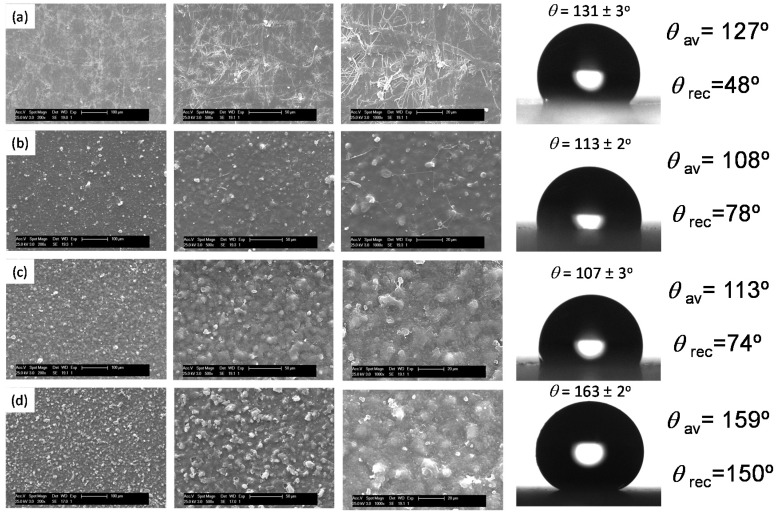
SEM images and contact angles of the surfaces obtained upon spraying (**a**) PS; (**b**) a 50/50 wt % blend of PS/PS-*co*-P5FS; (**c**) a 25/75 wt % blend of PS/PS-*co*-P5FS and finally (**d**) the pure PS-*co*-P5FS. The sprayed solutions have a total concentration of 50 mg/mL solutions. The number of layers deposed is 10. θ_av_ = advancing contact angle, θ_rec_ = receding contact angle.

In order to further increase the surface hydrophobicity, instead of adding an inorganic hydrophobic charge, blends of PS with the PS-*co*-P5FS copolymer were employed at high concentration (50 mg/mL) to fabricate microstructured surfaces. The SEM images of the surface structures and the resulting contact angle measurements are depicted in Figure 5. The incorporation of 50 wt % of PS-*co*-P5FS significantly alters the microstructure observed previously of pure PS. Instead of fiber-like structures, a corpuscular morphology with micrometer size microspheres (5–10 μm in diameter) partially embedded in a polymer matrix can be observed. Moreover, an increase of the amount of PS-*co*-P5FS within the blend to 75%, or even using only the copolymer, leads to surfaces with an increasing density of microspheres. As a result, the surface properties changed accordingly. PS surfaces form fibrous microstructures with contact angles of around 130°. The incorporation of the fluorinated copolymer changed the morphology and the wettability. In contrast to what would be expected by the incorporation of a fluorinated polymer, the contact angle decreases to values of around 110°. Most probably, the topographical changes with a decrease of the specific surface area and a Wenzel wetting regime can, at least to some extent, explain this observation. However, in the case of using the copolymer, the surface becomes superhydrophobic and exhibits contact angles above 160°. In this case, the increasing density of topographic features of these textured coatings promotes the transition between a Wenzel to a Cassie–Baxter state. Thus, microscopic pockets of air are trapped within the micro-texture, resulting in both high contact angles as well as low roll off angles (in our case below 10°).

### 2.3. Fabrication of Gradient Surface Patterns

**Figure 6:**
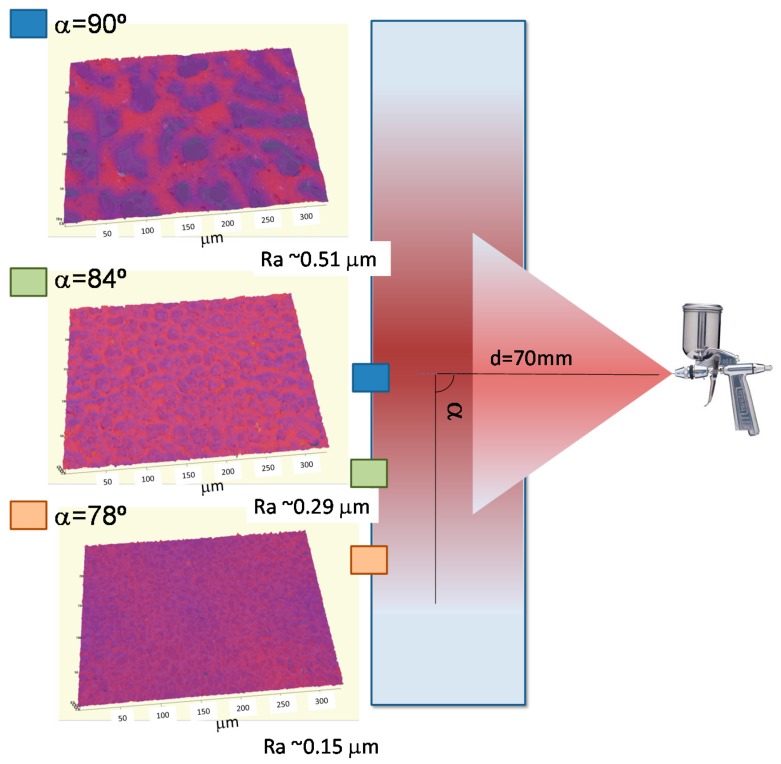
Schematic of spray apparatus employed to spray PS and blends of block copolymers and PS. The samples were sprayed at a distance d = 7 cm and pressure P = 28 Psi, under a constant temperature, to create five layers. The color images correspond to the 3D images of the samples at different positions from the center to the edge of the sprayed area.

Previous studies on the use of spray to fabricate gradient surfaces focused on the variation of the distance between the jet and the substrate [26]. Herein, we will explore the variation of the surface morphology as a function of the spraying angle. For this purpose, the fabrication of gradient surfaces by spraying was carried out applying the polymer solution in the center of a glass slide as depicted in Figure 6. To illustrate the preparation of gradient surfaces, diluted solutions of 5 mg/mL and deposing five layers were employed. As evidenced by the 3D profile images depicted in Figure 6, the surface topography changes linearly from the center to the edge of the sample. In particular, the surface roughness in the center, *i.e.*, when the jet is perpendicular to the substrate (α = 90°) is rather high: R_a_ ~ 0.51 μm. However, surface roughness decreases gradually to R_a_ ~ 0.29 μm when the angle between the jet and the substrate is around 84° and finally becomes R_a_ ~ 0.15 μm in the edge of the sprayed area (angle of ~78°). As a result, the surface morphology clearly depends on the lateral distance. In the center of the sprayed region, a large amount of polymer solution comes in contact with the surface and, as has been mentioned in Section 2.1, this induces the formation of a smooth surface. On the contrary, towards the edge of the sprayed area, the amount of polymer decreases, the surface is only partially covered, and a microtextured pattern is observed.

### 2.4. Thermoresponsive Sprayed Surfaces

**Figure 7:**
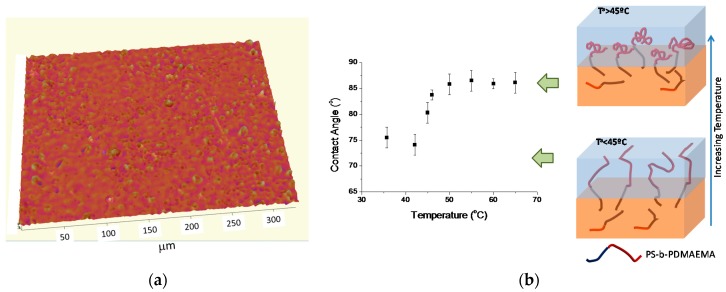
(**a**) Surface image obtained by using a 3D-optical profiler; (**b**) Variation of the contact angle as a function of the temperature on sprayed films composed of 50 wt % of PS and 50 wt % of PS-*b*-PDMAEMA. The polymer concentration employed was 50 mg/mL.

The thermal response of PDMAEMA both in solution and also on surfaces has been already described. Surfaces decorated with PDMAEMA have been, for instance, prepared by grafting-onto or grafting-from methodologies. However, independently of the strategy employed, these require the use of time-consuming multi-step procedures. Herein, we explored the formation of microstructured and thermal responsive surfaces by incorporating a PS-*b*-PDMAEMA block copolymer into a PS homopolymer matrix. The 3D optical profile image depicted in Figure 7 indicated the formation of a micropatterned surfaces with R_a_ ~ 0.59.

The surface coatings were prepared from blends of PS and PS-*b*-PDMAEMA using 50 mg/mL sprayed onto a glass substrate. In order to study the thermoresponsive behavior of the surface, contact angle measurements were carried out by heating the substrate between 35 and 65 °C and measuring the contact angle at different temperatures. As depicted in Figure 7, the contact angle below the Low Critical Solution temperature (LCST), *i.e.*, below 40 °C remains in values of around 75°. However, above 40 °C, the contact angle gradually increased from 75° up to 87°. Finally, the contact angles were observed at 50 °C, and, upon further heating, remained constant. Thus, an increase of the hydrophobicity indicated the formation of intrachain hydrogen bonding rather than interchain H-bonding observed below the LCST. It is worth mentioning that the LCST value observed is in good agreement with previous studies carried out on block copolymers in solution [27] in which LCST values between 45 and 50 °C were observed for similar block copolymer compositions.

## 3. Experimental Section

### 3.1. Materials

Styrene (S, Aldrich, Darmstad, Germany) and dimethylaminoethyl methacrylate (DMAEMA, Aldrich, Darmstadt, Germany) were purified by reduced pressure distillation to remove inhibitor. The monomers were stored at −5 °C for later use. Benzyl bromide (AR) was normally distilled and stored under an argon atmosphere at −5 °C. CuBr and 2,2′-bipyridyl were used as received without further purification. *N*,*N*,*N*′,*N*″,*N*″-pentamethyldiethylenetriamine (PMDETA) (Aldrich, 99%), copper (I) bromide (CuBr) (Aldrich, 98%), ethyl-2-bromoisobutyrate (EBrIB), 2,3,4,5,6-pentafluorostyrene, poly(ethylene glycol) methyl ether methacrylate and the rest of solvents were employed as received without further purification. In this study, glass covers (0.15 mm thickness, Menzel-Glaser, Braunschweig, Germany) were employed as supports.

### 3.2. Polymer Synthesis

The copolymers and block copolymers used throughout this work were prepared by Atom Transfer Radical Polymerization (ATRP) in order to obtain low polydispersity and controlled chain length.

#### 3.2.1. Preparation of Poly(2,3,4,5,6-pentafluorostyrene)-*co*-Polystyrene

The random copolymer of poly(2,3,4,5,6-pentafluorostyrene)-*co*-polystyrene p(PS-*co*-P5FS) was synthesized by Atom Transfer Radical Polymerization (ATRP). The polymerization was performed in Schlenk flasks previously flamed and dried under vacuum (this conditions are general for all the polymerizations). It was carried out using the following stoichiometry [M1]/[M2]/[I]/[CuBr]/[L] = 5:45:1:1:1 where M1 = PS, M2 = P5FS, I = EBrIB, L = PMDETA in toluene. The reaction mixture was degassed by three-pump-thaw cycles and placed in a thermostatic oil bath at 90 °C; after the polymerization, the mixtures were cooled to room temperature; and the contents were diluted with dichloromethane and passed through a neutral alumina column to remove the copper salt. After removing the solvent, the polymers were precipitated in hexane, washed and dried under vacuum (M_n_: 10,800 g/mol; Polydispersity (PD): 1.14). The final copolymer composition according to ^1^H-NMR was 10 mol % of PS and 90 mol % of P5FS.

#### 3.2.2. Synthesis of Polystyrene-*co*-poly[poly(ethylene glycol) Methyl Ether Methacrylate] (PS-*co*-PPEGMA)

The preparation of PS-*co*-PPEGMA, was carried out using the following stoichiometry [M1]/[M2]/[I]/[CuBr]/[L] = 48:12:1:1:1 where M1 = PS, M2 = PEGMA, I = EBrIB, L = PMDETA in toluene. The reaction mixture was degassed by three-pump-thaw cycles and placed in a thermostatic oil bath at 90 °C; after the polymerization, the mixtures were cooled to room temperature; the contents were diluted with dichloromethane and passed through a neutral alumina column to remove the copper salt. After removing the solvent, the polymers were precipitated in hexane, washed and dried under vacuum (M_n_: 6100 g/mol; PD: 1.2). The final polymer composition determined by ^1^H-NMR produced evidence of a copolymer formed by 65 mol % of PS and 35 mol% of PEGMA.

#### 3.2.3. Synthesis of Polystyrene-block-poly(dimethylaminoethyl Methacrylate) (PS-*b*-PDMAEMA)

In a typical polymerization experiment, 0.60 g (3.2 mmol) phenylethyl bromide, 0.56 g (3.2 mmol) *N*,*N*,*N*′,*N*″,*N*″-pentamethyldiethylenetriamine and 0.46 g (3.2 mmol) CuBr were placed in a dried 100 mL three-necked flask which was flushed with nitrogen. Pre-degassed styrene (20 g, 192 mmol) was added to the flask immersed in an oil bath at 85 °C, and then the solution was magnetically stirred for 4 h under a nitrogen atmosphere. Over this period, the originally red translucent polymeric solution turned dark and opaque. After the polymerization was completed, the polymer was diluted by 20 mL CHCl_3_, and then precipitated in excess methanol after passing through an alumina column. The white powder was purified by re-dissolution in CHCl_3_ and reprecipitation in methanol, and then dried at 60 °C under vacuum.

Synthesis of block copolymer: In a Schlenk tube, 0.819 g (0.182 mmol) PS-Br macroinitiator, 0.019 g (0.135 mmol) CuBr, and 0.023 g (0.135 mmol) *N*,*N*,*N*′,*N*″,*N*″-pentamethyldiethylenetriamine (PMDETA), 20 mL of pre-degassed DMF was introduced in a nitrogen atmosphere. The Schlenk was immersed in an oil bath at 90 °C and the ATRP was started by adding 2.55 g (16.2 mmol) of DMAEMA. The reaction was left for 24 h with continuous stirring. After the polymerization was completed, the former block was precipitated in methanol after passing through an alumina column, and dried at 60 °C under vacuum. According to ^1^H-NMR, the block copolymer has a composition of PS_43_-*b*-PDMAEMA_16_. Gel Permeation Chromatography (GPC) of the block copolymer carried out in Tetrahydrofuran (THF) produced a narrow polydispersity of 1.22 indicating the complete initiation of the polystyrene macroinitiator.

In addition, two different homopolystyrenes (PS) were employed. On the one hand, a rather short PS with 40 units was prepared by ATRP. On the other hand, commercially available high molecular weight polystyrene (Aldrich, M_w_ = 2.50 × 10^5^ g/mol, ~250 units) was used as polymeric matrix for the blend.

### 3.3. Film Preparation by Spraying

An air brush (Sealey AB931 with working pressure between 15 and 45 psi, Essex, UK) was connected to a compressed air tank (pressure P = 28 psi) to spray coat the polymer solution at a distance of ~7 cm onto the substrate. The air brush was moved horizontally during the spraying process. The diameter covered by the conical spray jet at the substrate over the duration of the spraying was ~3 cm in diameter, while the size of the glass slide was 8 cm × 2 cm.

Two spray-deposition modes were employed. The first mode involves one, five, and 10 cycles fixed in the center of the glass slide. In the second mode, the deposition was carried out over the entire film using one, five, and 10 cycles. The polymers and their blends were dissolved in chloroform at a variable concentration between 5 and 50 mg/mL.

### 3.4. Characterization

^1^H-NMR spectra of polymers were recorded at 70 °C in deuterated dimethyl sulfoxide (DMSO-*d*_6_) or in CDCl_3_ with a Bruker Advance spectrometer (Billerica, MA‎, USA) operating at 300 MHz. The proton spectra were used to determine the conversion, composition and modification degree of the different synthesized copolymers. The number-average molecular weight (M_n_), weight-average molecular weight (M_w_), and the polydispersity (M_w_/M_n_) were measured by size exclusion chromatography (SEC) with a chromatographic system (Waters Division Millipore, Madrid, Spain) equipped with a Waters model 410 refractive-index detector (Madrid, Spain). Dimethyl formamide (99.9%, Aldrich) containing 0.1% of LiBr was used as the eluent at a flow rate of 1 cm^3^/min at 50 °C. Styragel packed columns (HR2, HR3, and HR4, Waters Division Millipore) were employed. The calibration was performed with poly(methyl methacrylate) standards (Polymer Laboratories Ltd., Amherst, MA, USA) ranging from 2.4 × 10^6^ to 9.7 × 10^2^ g/mol.

Optical Profilometry: Analysis of the wrinkle formation as well as the cross-sectional profiles were obtained by using a Zeta-20 optical profiler (Zeta Instruments, San Jose, CA, USA) with different optical objectives and with 13 nm vertical resolution. Arithmetic average of absolute values (R_a_) were obtained using the Zeta3D™ metrology systems (San Jose, CA, USA).

Scanning electron microscopy (SEM) micrographs were taken using a Philips XL30 (Amsterdam, The Netherlands) with an acceleration voltage of 25 kV. The samples were coated with gold-palladium (80/20) prior to scanning.

## 4. Conclusions

In this manuscript, based on polymer blends and copolymers bearing different functional groups, we highlighted the versatility of the spray methodology to prepare microstructured surfaces with variable surface properties.

The number of layers applied, but overall the polymer concentration, clearly influenced the final surface topography. Whereas low and high concentrations led to surfaces with micropatterns, the spray of intermediate concentrations 25–30 mg/mL produced rather smooth surfaces.

Together with the concentration, the blend composition, and, in particular, the chemical structure of the copolymer employed also induced significant changes on the final morphology. Whereas spray of high concentration PS solutions formed fibrous morphologies, the addition of fluorinated polystyrene dramatically changed the morphology observed. Instead of fibers, a corpuscular morphology composed of micrometer size particles was found. These changes have an effect also on the surface wettability. As a result, a change in the wetting regime from a Wenzel to a Cassie–Baxter allowed us to fabricate superhydrophobic surfaces.

Finally, two other aspects have been considered in this manuscript. On the one hand, we provided evidence of the formation of a surface with gradient surface morphology by spraying in the center of a support. On the other hand, the use of stimuli-responsive polymers such as PDMAEMA allows us to obtain more sophisticated interfaces in which the wettability can be altered as a function of environmental parameters such as the temperature.

In summary, using the appropriate blend composition and polymer concentration, a simple spraying technique enables the fabrication of surfaces with different microtextures that may be employed in multiple applications ranging from antifouling or non-adherent surfaces to biocompatible or adhesive surfaces.
